# Fucoidans: Downstream Processes and Recent Applications

**DOI:** 10.3390/md18030170

**Published:** 2020-03-18

**Authors:** Ahmed Zayed, Roland Ulber

**Affiliations:** 1 Institute of Bioprocess Engineering, Technical University of Kaiserslautern, Gottlieb-Daimler-Straße 49, 67663 Kaiserslautern, Germany; ahmed.zayed1@pharm.tanta.edu.eg; 2 Department of Pharmacognosy, Tanta University, College of Pharmacy, El Guish Street, Tanta 31527, Egypt

**Keywords:** fucoidans, extraction, brown algae, production, bioactivities

## Abstract

Fucoidans are multifunctional marine macromolecules that are subjected to numerous and various downstream processes during their production. These processes were considered the most important abiotic factors affecting fucoidan chemical skeletons, quality, physicochemical properties, biological properties and industrial applications. Since a universal protocol for fucoidans production has not been established yet, all the currently used processes were presented and justified. The current article complements our previous articles in the fucoidans field, provides an updated overview regarding the different downstream processes, including pre-treatment, extraction, purification and enzymatic modification processes, and shows the recent non-traditional applications of fucoidans in relation to their characters.

## 1. Introduction

Polysaccharides, nucleic acids, and peptides are considered the main three types of bioactive polymeric macromolecules [1]. Among these, polysaccharides serve various roles in living cells including structural functions, where cellulose and chitin represent the major components of the different cell wall matrices [2,3], energy storage (e.g., starch and glycogen) [4,5], and hydration and signaling functions (e.g., mucilage and alginic acid) [6,7].

Particularly, marine homo- and heteropolysaccharides are derived from marine organisms, which represent a large part of global biodiversity [8]. Among these are the algal polysaccharides, such as fucoidan and alginate in brown seaweeds, carrageenan in red seaweeds and ulvan in green seaweeds. These were reported to have interesting nutraceutical, biomedical, pharmaceutical and cosmeceutical applications, including dietary fibers; anti-inflammatory, anti-tumor, anti-oxidant, hepatoprotective and anti-coagulant properties; and drug carrier functionality. Therefore, they have been extensively investigated during the last few decades [9,10,11,12,13], especially after the emergence of glycobiology and glycomics [14,15,16,17].

Polysaccharides such as dietary fibers of brown algae are abundant and diverse (e.g., alginates, cellulose, fucoidans and laminarins) constituting the major components (up to 75%) of the dried thallus weight (% DW) [18,19,20]. Previous work investigated their abundance in different species, reporting *Fucus*, *Ascophyllum*, *Saccharina*, and *Sargassum* to contain 65.7, 69.6, 57.8 and 67.8 % DW, respectively [21,22]. Specifically, fucoidans are found in the cell walls and extracellular matrices of brown algae in addition to more than 265 genera and 2040 species of marine invertebrates (e.g., sea cucumbers), where they perform vital structural functions [23,24,25,26]. Fucoidans are assumed to act as cross-linkers between the major threads of cellulose and hemicellulose, promoting cellular integrity and maintaining cellular hydration (especially during drought seasons) [27]. They also act in other reproductive, immune and cell-to-cell communicative roles [23]. As recommended by the International Union of Pure and Applied Chemistry (IUPAC), fucoidans is a general term used to describe sulfated L-fucose-based polymers including sulfated fucans cited by the Swedish scholar Kylin, as well as other fucose-rich sulfated heteropolysaccharides [23,28]. Their chemical structures, in terms of monomeric composition and branching, are quite simple in marine invertebrates compared to their analogues in brown algae [13,29].

Hundreds of articles have thoroughly discussed and reviewed the biological, pharmacological and pharmaceutical applications of fucoidans [30,31,32,33], including nanomedicine, [34] which has made it a hot topic in the last few decades [35,36,37]. All these studies tried to investigate fucoidans molecular mechanisms in relation to their chemical structure and physicochemical properties. Therefore, different hypotheses were suggested for each activity, such as anti-tumor [31,38,39,40], anti-coagulant [41,42], anti-viral [43,44] and anti-inflammatory activity[45,46]. These investigations revealed that various factors are relevant, such as molecular weight, sulfation pattern, sulfate content and monomeric composition [47,48,49]. For example, different fractions were produced with different physicochemical properties in our previous experiments; sulfation pattern and sulfate content were highly related to anti-viral and cytotoxic activities against HSV-1 and Caco-2 cell lines, respectively, while molecular weight and sugar composition were potential factors in anti-coagulation activity [41,50]. In addition, degree of purity was reported as an influential factor [32], where co-extracted contaminants (e.g., phlorotannins or polyphenols) could lead to significant interference in anti-oxidant activity and, consequently, cosmetic applications [51,52]. 

Therefore, several key production challenges regarding fucoidans were discussed in our last review article in order to obtain a product that follows the universal good manufactured practice (GMP) guidelines. The article discussed sources of heterogeneity in extracted fucoidans, including the different biotic (e.g., biogenic, geographical and seasonal factors) and abiotic (e.g., downstream processes) factors affecting the fucoidans physicochemical and chemical properties [53]. Others patented production techniques that have assisted in the marketing of several commercial fucoidans by well-known companies (e.g., Sigma-Aldrich^®^, Algues and Mer and Marinova^®^) derived from *Fucus vesiculosus* and other brown algae species [54,55,56].

Furthermore, the improvement of fucoidans activity was investigated, targeting several points. Among these was the modification of the chemical structure of the native fucoidans scaffolding, including depolymerization [57,58] and over-sulfation [59]. These modifications could be attempted chemically [60], enzymatically [35,61] or physically [62]. Predetermined synthesis of oligomers [63,64] and low molecular weight polymers with defined monomeric units [65] is also involved. Additionally, fractionation of fucoidans is a common approach during extraction and purifications steps by applying different extraction and purification conditions (e.g., pH, time, molarity of NaCl) [49,55]. 

The current article aimed at complementing our previously published article discussing the reasons for heterogeneity of fucoidans [53]. It reviewed and evaluated the different downstream processes used in production as the most important abiotic factors affecting the fucoidans quality and structural features; it then addressed recent uncommon applications and prospective bioproduction of fucoidans. In addition, the updated status of enzymatic structural modifications of fucoidans, especially by fucoidanases, were presented.

## 2. Global Market and Cultivation of Brown Algae

Marine hydrocolloids (e.g., agar, carrageenan and alginate) are of particular industrial interest, with worldwide annual production of approx. 100,000 tons and a value above US $1.1 billion [66]. Based on FAO periodical reports (FAO, 2014, 2016), among the top seven most-cultivated seaweeds, three taxa are mainly used for hydrocolloids production; these include Rhodophyta (e.g., *Eucheuma* sp. and *Kappaphycus alvarezii*) for carrageenan production and *Gracilaria* sp. for agar production [67]. These data encouraged the global marine market to escalate the production yield by finding alternative, eco-friendly seaweed cultivation techniques, such as sea farming or aquaculture and biotechnology [53]. In 2014, the annual production of cultivated seaweeds reached 27.3 million tons [68], representing 27% of the total marine aquaculture production, while the global market of marine biotechnology (blue biotechnology) for industrial applications has been expected to achieve US $4.8 billion in 2020 and grow to US $6.4 billion by 2025 [69].

**Figure 1:**
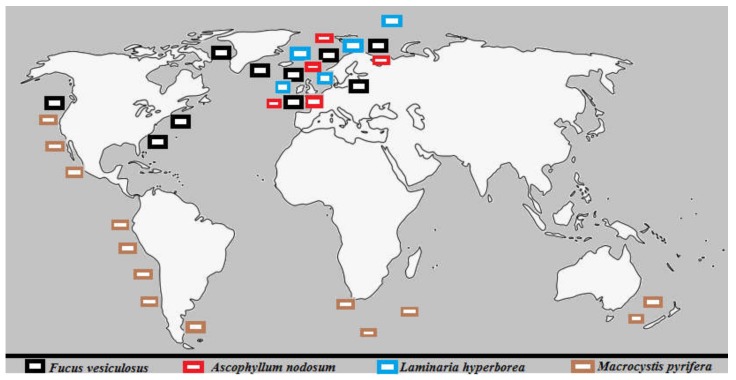
Global distribution of the major brown seaweeds’ species. They dominate tropical to temperate marine forests and intertidal regions.

Species of brown macroalgae (Phaeophyceae) are distributed among the orders Fucales and Laminariales, which are the major commercial sources of the algal sulfated polysaccharides, in addition to Chordariales, Dictyotales, Dictyosiphonales, Ectocarpales, and Scytosiphonales. Moreover, phylogenetic analysis showed that Fucales are one of the largest and most diversified orders within Phaeophyceae, having eight families (41 genera and 485 species), named Ascoseiraceae, Cystoseiraceae, Durvillaeaceae, Fucaceae, Hormosiraceae, Himanthaliaceae, Sargassaceae, and Seirococcaceae [70]. Figure 1 illustrates the distribution of several examples of well-known brown algae species which are considered potential sources of sulfated polysaccharides dominating tropical to temperate marine forests and intertidal regions. The data were based on Wahl, et al. [71].

Furthermore, like terrestrial plant tissue culture (PTC), several biotechnological attempts were performed to cultivate and/or regenerate thallus from different species of brown seaweeds using seaweeds tissue culture [72]. They include micropropagation, callus induction and protoplast isolation [69,73,74,75]. They are very promising techniques as it may not only help to overcome the previously mentioned fucoidans production heterogeneity challenges [53] but also provide a sustainable supply [76]. However, compared to PTC, STC is still not well-enough established to be used for production of hydrocolloids and fucoidans [77] or cultivation in closed, well-controlled bioreactors, as in case of the red algae organism *Agardhiella subulata* [78].

## 3. Downstream Processes

**Figure 2:**
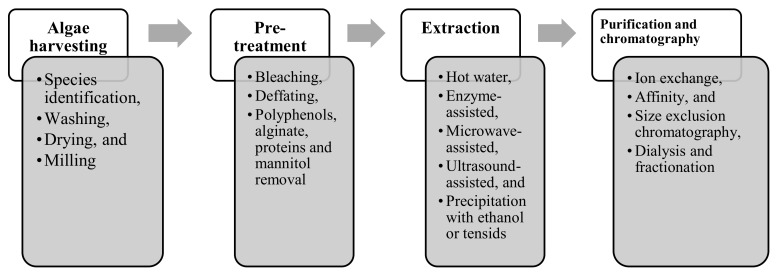
Required downstream processes including steps in each process for fucoidans production.

Fucoidans are anionic polymers occurring in highly complicated matrices in cell walls and intercellular spaces along with other carbohydrate polymers (e.g., alginate, cellulose and laminarin), polyphenols and proteins [79]. Additionally, due to the sulfate ester groups, fucoidans are water soluble polysaccharide polymers [80] exhibiting high affinity to other cell wall components, especially polyphenols [81]. Therefore, various and complicated downstream processes are required to remove such extraneous substances before and after precipitation with ethanol or cationic surfactants to obtain high-purity fucoidans [82,83]. The processes always include pre-treatment, extraction and purification stages as shown in Figure 2.

### 3.1. Pre-Treatment

After harvesting algal biomass from beaches, the biomass should be washed thoroughly with tap water to remove sands and epiphytes, then dried and milled to increase the area-to-mass ratio. Several pre-treatment steps are performed before the extraction step to release fucoidans from intercalating components, ease the following extraction process, improve the extraction yield, and decrease the possible interferences from co-extracted components in purification and biological investigations. 

Previous experiments tried to remove pigments (e.g., chlorophyll, flavins and carotenoids) and lipids in specific bleaching and defatting steps with acetone, toluene, charcoal or 80%–85% (*v/v*) ethanol [34,84,85]. Since fucoidans are negatively charged molecules, they remained unaffected by incubation with organic solvents (e.g., acetone, toluene or hexane:isopropanol (3:2) mixture) during pre-treatment of the dried algal biomass. Such extracts were further treated to obtain carotenoids, represented by fucoxanthin in brown algae [86], lipids and fatty acid metabolites (especially essential polyunsaturated fatty acids (PUFA) and fucosterol), adding to nutraceutical applications of brown algae [87,88]. In contrast, activated carbon materials, such as charcoal, adsorb the target fucoidans molecules, adversely affecting the final production yield [79]. 

Other studies tried to exclude the tightly non-covalently bound polyphenolic compounds represented by phloroglucinol-type phlorotannins [89], which contribute to the light to dark brown color of the crude fucoidans extract (along with fucoxanthin) [41,81]. They reported comparatively high phlorotannins content, reaching approximately one fifth of the brown algae dry weight [25]. Phlorotannins perform major structural and physiological functions, like tannins found in plants, including defense against biotic and abiotic stresses [90,91]. Despite of the great pharmacological importance of phlorotannins [92,93], their presence in high-quality fucoidans is not acceptable because of the possibility of interference with the anti-oxidant [25,52,94] and anti-tumor activities of fucoidans [95]. Therefore, the natural phenolics content of fucoidans should be determined before the measurement of their biological activities [96]. Therefore, nearly all pre-extraction protocols for fucoidans involved strategies to remove such contaminants, e.g., incubation with EtOH:H_2_O:HCHO (16:3:1) (*v*/*v*/*v*) at pH 2. Under such conditions, formaldehyde enhances the crosslinking and polymerization of such polyphenolic contaminants and the high volume of ethanol results in protein denaturation [41,60,97,98]. However, the toxicity of formaldehyde limits its utilization in pre-treatment protocols [51].

**Figure 3:**
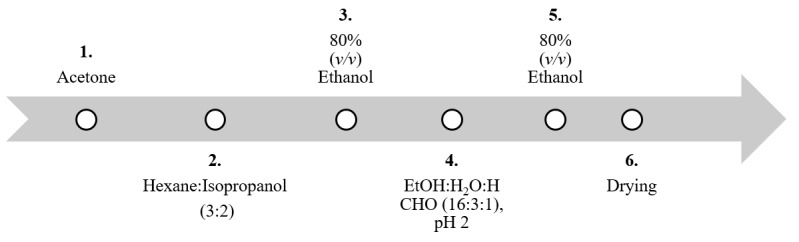
Overview of optimized pre-treatment steps of the dried algae biomass before fucoidans extraction. All steps were performed at 25 °C overnight and the ratio between dried algal biomass to solvent was 1:10, except for the acetone step, which was 1:20 (modified after [98,102]).

Furthermore, pre-treatment steps are performed to remove other carbohydrates such as alginate, the major hydrocolloids in brown algae [99]. This is commonly removed by formation of water-insoluble calcium complex either before [60] or during the extraction procedure using 1%–4% (*w/v*) CaCl_2_ followed by a filtration or centrifugation step to remove the formed precipitate [58,98,100,101]. These previously mentioned procedures were optimized using successive incubation, centrifugation or filtration, washing and drying for the main extraction step of the dried, milled algal biomass, as described in Figure 3. The application of such an optimized protocol resulted in a dried, pre-treated powder representing 71% (*w/w*) of the starting material [98]. Despite these results, downstream processing of fucoidans, except with enzymatic modification, starts with a small scale (e.g., 5–10 g of the dried algal biomass) to optimize parameters like dried biomass to solvent ratio, temperatures, pH, and incubation time, based on preliminary quality and yield of crude fucoidans measured by infra-red spectroscopy (IR), simple sugar tests and elemental analysis. After this, transfer to large scale production could be accomplished using larger biomass quantities (e.g., 500–1000 g).

Due to several complicated pre-treatment steps, general protocols always employ a single incubation step using the ternary mixture composed of CH_3_OH:CH_3_Cl:H_2_O (4:2:1) (v/*v*/*v*) [103], binary mixture of CH_2_Cl_2_:EtOH (94.2:5.8, *v/v*) [104], or aqueous ethanol (e.g., 95% *v/v*) [105,106] to remove pigments [107], polyphenols [51,103] and lipids [108]. Nevertheless, pre-treatment steps may be insufficient for complete removal or prevention of some residual co-extraction. 

Notably, all these procedures were carried out at room temperature in organic solvents and high volumes of ethanol, in which fucoidans are insoluble. Theoretically, the native structural backbone should not be affected. However, similar polymeric carbohydrates such as laminarin may still be present, contaminating the extract after these steps. 

Recently, in order to decrease pollution of organic toxic solvents, compressional-puffing pre-treatment was applied for *Sargassum hemiphyllum* and *S. glaucescens* fucoidans. The pre-treatment method was based on mechanical pressure at higher temperatures that loosen the cell wall matrix before the step of extraction. Such methods succeeded in increasing the production yield, but they affected the molecular features of the fucoidans, including molecular weight [109,110].

### 3.2. Extraction 

As previously mentioned, fucoidans are principally anionic water-soluble macromolecules. Therefore, they can be extracted from the pre-treated biomass using a simple hot- or cold-water incubation. Afterwards, the extracted fucoidans can be precipitated by high volumes of solvents with a low dielectric constant (e.g., >70% (*v/v*), > 2.5 volume ethanol [111,112], <2 volume acetone [113]) or cationic surfactants (e.g., hexadecyltrimethylammonium bromide (Cetavlon^®^) 10% (*v/v*)) [55] via an affinity complex formation at low temperatures (4 °C) to remove the undesired salts from the sulfated polysaccharides [52]. This specific precipitation reaction between fucoidans and Cetavlon^®^ is applied in screening tests of microorganisms for putative fucoidanase activity [114].

Ale et al. published comprehensive articles discussing the history of extraction, including the different classical extraction methods of fucoidans, and reported that extraction procedures significantly affect the polymers monomeric composition, even for the same organism [60,115]. Beyond simple hot water extraction [58,116], attempts were made to increase the selectivity and extraction yields, including extraction in acidic [117], alkaline [118], and buffered [41,119] aqueous solutions. However, a neutralization step is required, using Na_2_CO_3_ or (NH_4_)_2_CO_3,_ directly after extraction to guard against the non-specific acidic hydrolysis of the polymer [101,115]. Such drastic pH changes affect the chemical and physicochemical properties of fucoidans during the extraction step.

Currently, besides the previously discussed classical extraction methods based on thermal energy, extraction protocols based on vibrational energy have been developed. These protocols are based on microwave-assisted (MAE) [120,121] or ultrasound-assisted (UAE) [94,122] extraction steps to elicit cell wall degradation which improves the polymer release into aqueous solvent. These protocols were optimized either using an approach that modified one factor at a time or a multiple factorial design, setting the polymers production yield, monomeric composition and biological activities as the measured responses.

Recently, combined sulfated polysaccharides extraction protocols were optimized from different brown algae species using hydrothermal-assisted extraction (HAE) followed by sequential ultrasound and thermal technologies [123]. Similarly, subcritical water extraction was applied to increase the production yield of fucoidans from *Nizamuddinia zanardinii* [124]; such mild conditions may be advantageous to preserve the native chemical backbone and physicochemical characters of fucoidans. 

Recently, as a trial to reduce such undesirable effects, enzyme-aided or assisted extraction (EAE) protocols are being developed using enzymes instead of harsh chemicals and high extraction temperatures during extraction. These include cellulase, papain, laminarinase, alginate lyase, and protease, which are present in products of Novozymes [79,125,126,127,128]. In addition, other cost-effective and time-saving techniques are reported, like those for terrestrial plant polysaccharides, such as extraction under vacuum to lower the boiling point of water and hence avoid possible heat-induced fucoidans degradation [129]. Alternatively, 0.5% (*w/v*) ethylenediaminetetraacetic acid (EDTA) was applied at 70 °C for simultaneous extraction of *Laminaria japonica* fucoidans and removal of pigments [130].

### 3.3. Separation Physical Methods

Filtration, dialysis and centrifugation, either for the algal biomass or precipitates, are also among the downstream processes after pre-treatment and extraction steps [131,132,133]. Cross-flow filtration and dialysis against water are usually performed using different molecular weight cut-off (MWCO) membranes for isolation of fucoidans from smaller compounds depending on the high molecular weight of fucoidans [134] and also for fractionation purposes, where low molecular weight fucoidans (LMWF) can be separated from high molecular weight analogues (HMWF) [49].

In addition, filtration, concentration, and fractionation are simultaneously performed using centrifugal concentrators (Vivaspin^®^) equipped with membranes with certain MWCO, like in protein purification. However, in some cases, especially in the presence of bulk masses or high concentrations of salts and small contaminants, the use of centrifugal concentrators becomes practically and economically unsuitable for fucoidans purification. In such cases, bulky contaminants result in membrane clogging leading to its deterioration and increasing the production cost.

### 3.4. Purification

Despite the previously mentioned purification steps, residuals of co-extracted contaminants are still present, and resulting fucoidans are still crude-type. [27]. Therefore, further selective purification steps are needed to obtain a high-quality product for reproducible and accurate biological investigations. Some researches adopted simple, non-chromatographic steps, such as bleaching of the crude fucoidans (NaClO_2_ in dilute HCl) followed by precipitation with cetyltrimethylammonium bromide [135] or by cold overnight incubation in aqueous buffered solution of calcium acetate (20 mM, pH = 6.5 -7.5) followed by dialysis [136]. In addition, membrane filtration was reported to produce fucoidans fractions of different molecular weight [137].

However, other chromatographic purification techniques were discussed in our previous publications [41,53,98,102]. Almost all the chromatographic techniques are based on the permanent negatively charged sulfate ester groups distributed on the polymer backbone which allow selective fucoidans capture. However, carboxylated (e.g., alginate) and phosphorylated (e.g., nucleic acids) compounds might interfere [138,139]. Therefore, the pH value of the applied solvents is critical during chromatographic purification. One option for this uses anionic exchange resins (e.g., diethylaminoethyl cellulose or DEAE-cellulose), which was performed at pH 7.2 using 0.1 M sodium phosphate buffer [140]. An alternative is cationic dyes (e.g., toluidine blue- or perylene diimide derivative), modified resins or chitosan functioning in buffered solutions [27,102]. Both anion exchange and dye affinity chromatography involve the use of highly concentrated NaCl elution solvents. As a result, a subsequent purification step using chromatographic gel permeation [141] or dialysis [140] is required to remove salts, increasing the production costs. Other methods based on the use of biological macromolecules, such as lectins and anti-thrombin III, were also reported [53].

Novel innovative purification techniques were recently developed, such as selective solid phase extraction for purifying fucoidans and other complex seaweeds polymers by molecularly imprinted polymers (MIP) [142,143] or MIP modified by deep eutectic solvents [142,143]. Abdella et al., developed a green and time-saving purification protocol using genipin cross-linked toluidine blue immobilized-chitosan beads employing fucoidans affinity to cationic thiazine dyes [102]. 

## 4. Recent Uncommon Applications

In addition to the classical therapeutic applications of fucoidans, including anti-coagulant [41,144], anti-viral [145,146], anti-inflammatory [46,147] and selective cytotoxic and anti-tumor uses [39,50], uncommon bioactivities, including cosmeceutical, pharmaceutical, diagnostic, and synergistic therapeutic applications were recently reported [32]. Recent fucoidans uses included therapeutic treatment of major blindness diseases [148]. It has also been used as a drug carrier, especially for anti-cancer treatments and anti-biotics. Additionally, fucoidans have been shown to improve drug bioavailability and efficacy in pharmaceutical formulations, including in nanoparticles, liposomes, microparticles, and semisolid formulations [28,149,150]. Table 1 summarizes some of the recent and uncommon fucoidans applications based on in-vitro or in-vivo studies, in addition to biogenic resources and physicochemical features. 

## 5. Enzymatic Modification of Native Fucoidans

Owing to their high molecular weight, therapeutic applications of native fucoidans face many challenges including structure elucidation, solubility, manufacturing, and handling [63,116], in addition to safety as a food supplement [175]. Structure elucidation and quantitation of native fucoidans is highly complicated and requires advanced or hyphenated spectroscopic techniques such asHPLC-MS/MS as it applied in Sea Cucumbers fucoidans [176,177]. Also, these techniques must be applied after a step of enzymatic or acid hydrolysis to transform the fucoidans polymers to oligomers. According to their molecular weight, fucoidans are classified into three classes: LMWF (<10 kDa), medium molecular weight fucoidan (MMWF) (10–10000 kDa), and HMWF (>10000 kDa) [31]. LMWF demonstrated better bioavailability and bioactivities than HMWF [178,179]. As a consequence, several articles reported physical, chemical and enzymatic modification of the native HMWF to get LMWF of higher biological activity [62]. Specifically, enzymatic modification of macroalgal polysaccharides, including fucoidans by either fucoidanases or sulfatases, is characterized by regioselectivity and stereospecificity. This new trend is considered crucial and highly promising for current and future applications of polysaccharides [180]. 

Nevertheless, our publications in 2009 particularly reviewed the specific enzymatic degradation of fucoidans induced by fucoidanases (EC 3.2.1.44) and α-_L_-fucosidases (EC 3.2.1.51), mainly those isolated from marine bacteria [35]. Cumashi, et al. studied the chemical structures of different fucoidans isolated from a number of brown algal species [181]. Their proposed models, which were highly appreciated and recommended by many researchers [60], showed the backbone of fucoidans to be mainly an alternating α-(1-4) and α-(1-3) linked _L_-fucopyranoside. Regarding the sulfation pattern, C-2 is usually substituted with sulfate ester groups in addition to alternating C-3 or C-4 in L-fucopyranose residue, according to the glycosidic linkages. In addition, branched chain polymers were also found as in *F. serratus*. Other minor sugar units (e.g., mannose, galactose, glucose and xylose) occur as well in fucoidans structure; however, their distribution pattern and positions are still unknown [60,181]. Now, the mechanism of enzymatic degradation can be described in relation to fucoidans chemical structures.

Despite the increasing number of publications investigating fucoidanase activity of different marine species cell extracts, few of these enzymes have been isolated and characterized. Moreover, genome sequences encoding few fucoidanases have been published, including Ffa2 and FFA1 from *Formosa algae* KMM 3553^T^ [182,183], FcnA from *Mariniflexili fucanivorans* SW5T [184]. Therefore, specificity of fucoidanases, type of cleaved glycoside bond, structure-activity relationship studies and enzyme stability are still poorly described. It was only observed that identified microbial fucoidanses act only on fucoidans isolated from their respective symbionts [185]. In fact, fucoidanases have not actually been fully utilized yet as a powerful tool either for the structural studies of fucoidans or production of defined and well-characterized bioactive fragments of extracted fucoidans, as shown in Table 2.

Similarly, recent advances in bioinformatics and genome sequencing of microbial species have resulted in a continual increase of novel genome sequences. These genomes demonstrated various potential genes encoding for enzymes with biopolymer-degrading capabilities, such as *Shewanella violacea* DSS12 (NC_014012.1), *Formosa algae* KMM 3553 (NZ_LMAK01000014.1) [182], *Formosa haliotis* MA1 (NZ_BDEL01000001.1) [198], *Wenyingzhuangia fucanilytica* CZ1127 (NZ_CP014224.1) [199] and *Pseudoalteromonas* sp. strain A601 (MXQF01000000) [200]. Moreover, production of stabilized fucoidanases has been achieved by targeted truncation of the C-terminal of FcnA2, Fda1 and Fda2. This recently developed method may help with enzymatic production of defined degrees of polymerization and more bioactive products from native fucoidan substrates [201].

## 6. Conclusion and Future Prospective

As multifunctional molecules, fucoidans have received special interest based on their proven efficacy in different fields. The current article reviewed many aspects related to fucoidans’ production, mainly from brown algae. Biogenic source and downstream processes were shown as major factors determining their application, which is affected by molecular weight and quality grade of fucoidans. Therefore, the alteration of fucoidans’ native structure was recommended, especially as performed by fucoidanases. Their production in nanoform or in combination with other polymers can improve or modify their potential uses, allowing their expanded potential as therapeutic agents, e.g., in anti-cancer applications [202].

Production of high-quality purified fucoidans is urgently required to clarify the relationships between chemical structure and the various bioactivities attributed to fucoidans, eliminating any interference from contaminants. However, it was observed in some cases that crude extracts and presence of co-extracted contaminants, especially polyphenolic phlorotannins, have advantageous cosmeceutical effects due to their powerful anti-oxidant activity [203,204].

Novel techniques, either in cultivation or downstream processes, have been established, increasing the global production yields and reducing ecological and economic problems. A new advance toward achieving such goals was established by optimization of water extraction via measurement of kinetic parameters [205]. In addition to this, it is expected that most future trends in marine biotechnology research will focus on the cell wall and extracellular matrix components of brown algae, including fucoidans’ biosynthetic genes and production regulators [23,53,63,206,207,208]. Such trials may enable the scientific community to produce more bioactive molecules of fucoidans with defined characteristics, including degree of polymerization, sulfate content and pattern, in reproducible manners.

## Figures and Tables

**Table 1:** Some selected recent therapeutic, diagnostic and pharmaceutical applications of fucoidans including the biogenic sources.

Application	Biogenic Source	Quality Grade/Purification Method	Structural Features	Involved Mechanism	Ref.
**Therapeutic**					
Anti-viral (IAV)	*Kjellmaniella crassifolia* (Laminariales)	*		Inhibition of the viral neuraminidase (NA) Interference with the cellular EGFR pathway	[43]
Anti-metabolic syndrome	*Fucus vesiculosus* (Fucales)	Dialysis of crude alginate-free fucoidans	Alternating α(1→3)/α(1→4)linked fucose, Mw > 7.0 × 10^3^ g/mol	Regulation of jnk, akt, and ampk signaling Alleviation of insulin resistance Regulation of lipid metabolism	[151]
Anti-leishmaniasis		Commercial product purchased from Sigma-Aldrich^®^	Polymer of α-(1→3) linked fucose	Activation of the mitogen-activated protein kinase (MAPK)/NF-κB pathway against *Leishmania donovani*-infected macrophages	[152]
				Enhancement of dendritic cells maturation, production of pro-inflammatory cytokines, and down-regulation of anti-inflammatory cytokines	[153,154]
Immunostimulant	*Nizamuddinia zanardinii* (Fucales)	A fraction of DEAE Sepharose Fast Flow column	Highly branched polymer Mw: 953.6 × 10^3^ g/mol	Stimulation of RAW264.7 murine macrophage and NK cells	[155]
Anti-metastasis	*Undaria pinnatifida* (Laminariales)	DEAE-cellulose, and Sephadex G-100 column chromatography (purity>90%)	Mw: of 10.4356 × 10^4^ g/mol	- Suppression of Hca-F cell growth, adhesion, invasion, and metastasis capabilities, - Inactivation of the NF-κB pathway	[156]
Gastrointestinal tract protective		Purity ≥ 95% (Commercial product purchased from Sigma-Aldrich^®^)		Protection against H_2_O_2_-induced damage via activation of the NRF2 signaling pathway	[157]
Anti-malaria		- Partial purification by cetylpyridinum chloride Fractionation by DEAE-Sephadex A-25 column	Sugar monomers, and uronic acid, M.wt: approx. 15 × 10^3^ g/mol	In-vitro and in-vivo inhibition of erythrocytes invasion by *P. falciparum* merozoites	[158]
Renal protective	*Laminaria japonica* (Laminariales)		LMWF (Mw: 7 x 10^3^ g/mol)	Inhibition of overexpression of pro-inflammatory and pro-fibrotic factors, oxidative stress and apoptosis	[159,160]
Cardio-, hepatic- and renal protective		Commercial product purchased from Absunutrix Lyfetrition^®^		Reduction of oxidative stress, pro-inflammatory effects and injuries to the cardiac, hepatic, and renal tissues	[161]
Inhibition of tumor angiogenesis	*Sargassum hemiphyllum* (Fucales)	Hydrolyzed crude extract	LMWF; 760 g/mol	Suppression of HIF-1/VEGF-regulated signaling pathway	[162]
Pro-angiogenic	*Ascophyllum nodosum* (Fucales)	Fractionated with dialysis commercial crude fucoidan (ASPHY)	LMWF (<4.9 x 10^3^ g/mol)	Increase of the vascular network formation regulated via Erk1/2 and PI3K/AKT cell signaling pathways	[163]
Alleviation of diabetic complications	*S. Fusiforme* (Fucales)	Crude extract	Mw: 205.89x10^3^ g/mol, high sulfate content	- Suppression of oxidative stress - Alteration of the gut microbiota - Attenuation of the pathological changes in heart and liver	[164]
**Diagnostic**					
Imaging of cardiovascular diseases	*Ascophyllum nodosum* (Fucales)	An oxidative-reductive degraded crude extract (purchased from Algues and Mer, Ascophyscient^®^)	GMP-grade LMWF (7.1x10^3^ g/mol)	Synthesis of technetium-99m-fucoidan radiotracer for detection of P-selectin	[56]
		Commercial product from Algues and Mer		Synthesis of polycyanoacrylate-fucoidan microcapsules (Fuco-MCs) for detection of P-selectin	[165]
**Cosmeceutical**					
Anti-Photoaging	*Ecklonia cava* (Laminariales)	Enzymatic degradation of a commercial HMWF	LMWF (Mw: 8 × 10^3^ g/mol)	Anti-oxidant, anti-apoptotic, and MMP-9-inhibiting effects	[166]
Skin brightening and age spot reduction	*F. vesiculosus* (Fucales)	Crude extracts purchased from Marinova^®^ Pty Ltd.	58.6% fucoidans, 33.7% polyphenol	Increase of Sirtuin 1 (*SIRT1*) expression in vitro	[167,168]
Skin immunity, soothing and protection	*U. pinnatifida* (Laminariales)		89.6% fucoidans, <2% polyphenol		
Reconstruction of skin	*F. vesiculosus* (Fucales)	Commercial product from Sigma-Aldrich^®^ (not determined the degree of purity)		Increase of proliferating cell nuclear antigen (PCNA) p63 and α6-integrin expression	[169]
**Pharmaceutical technology**					
As vehicle for drug delivery	*F. vesiculosus* (Fucales)	Commercial product purchased from Sigma-Aldrich^®^	Mw: 57.26 ×10^3^ g/mol	- Chitosan-fucoidans-based nanoparticles for delivery of anti-cancers (e.g., curcumin-loaded NPs) - Nanoencapsulation of poly _L_-lysine	[170,171]
				Piperlongumine (PL)-loaded chitosan-fucoidan nanoparticles (PL-CS-F NPs)	[172]
				Synthesis of fucoidan/trimethylchitosan nanoparticles (FUC-TMC-NPs) as adjuvant in anthrax vaccine adsorbed	[173]
Green synthesis of silver nanoparticles				Synthesis of chitosan-fucoidan complex-coated AgNPs	[174]

*: Not specified.

**Table 2:** Source of fucoidans as a substrate and mode of action of some fucoidanases.

Biogenic Source of Fucoidans	Fucoidanase Source	Mode of Action	Ref.
*F. evanescens*	*Formosa algae* KMM 3553	Endo α-1→4	[61,182]
	*Pseudoalteromonas citrea* strains KMM 3296, KMM 3297, KMM 3298	Endo α-1→3	[186]
*F. vesiculosus*	*Dendryphiella. arenaria* TM94	Endo n.d. *	[187]
*Kjellmaniella crassifolia*	*Fucobacter marina* SA-0082	Endo β-1→4	[188]
*Cladosiphon okamuranus*	*Fucophilus fucoidanolyticus* SI-1234	Endo α-1→3	[189]
	*Flavobacterium* sp. F-31	Endo n.d.	[190]
*F. distichus*	*Littorina kurila*	Endo α-1→3	[191]
*Pelvetia canaliculata*	*Mariniflexile fucanivorans* SW5^T^	Endo α-1→4	[184]
*Undara pinnatifida*	*Sphingomonas paucimobilis* PF-1	Endo n.d.	[192,193]
*Saccharina cichorioides*	*Pseudoalteromonas citrea* strains KMM 3296, KMM 3297, KMM 3298	Endo α-1→3	[186]
*Nemacystus decipiens*	*Mizuhopecten yessoensis*	Endo n.d.	[194]
*Ascophyllum nodosum*	*Pecten maximus*	Exo n.d.	[195,196]
*Thelenota ananas* (Wild sea cucumber)	*Wenyingzhuangia Fucanilytica*	Endo n.d.	[197]

* n.d.: not determined.
